# Metabolic Signatures and Diagnostic Strategies for Different Types of Encephalitis

**DOI:** 10.1002/cns.71014

**Published:** 2026-07-11

**Authors:** Yue Cui, Haitian Nan, Zhen Wang, Daiquan Gao, Hong Ye, Yunzhou Zhang, Junjie Li, Weibi Chen, Lin Wang, Jing Zhang, Liyong Wu

**Affiliations:** ^1^ Department of Neurology Xuanwu Hospital, Capital Medical University Beijing China; ^2^ National Clinical Research Center for Geriatric Diseases Beijing China

**Keywords:** autoimmune encephalitis, cerebrospinal fluid, diagnostic biomarker, metabolomics, viral encephalitis

## Abstract

**Aims:**

To characterize metabolic signatures associated with different encephalitis types and to develop differential diagnostic models based on metabolic dysregulation profiling.

**Methods:**

Untargeted metabolomics was performed on cerebrospinal fluid (CSF) from a prospective cohort of 232 patients classified into viral encephalitis (VE, *n* = 82), autoimmune encephalitis (AE, *n* = 63), other infectious encephalitis (OIE, *n* = 25), and non‐encephalitis control (NE, *n* = 62) groups, with follow‐up samples from 10 participants.

**Results:**

Compared with controls, taurine‐associated metabolites were consistently downregulated across the encephalitis groups. Arginine and proline metabolism were identified as commonly dysregulated pathways in the encephalitis groups. Highly similar metabolic network topologies were observed between VE and AE, despite different key node metabolites (lysophosphatidylcholine vs. acylcarnitine, respectively). Three diagnostic models developed to distinguish VE from other groups achieved an area under the receiver operating characteristic curve > 0.8 in the training and validation sets. Glial fibrillary acidic protein antibody‐positive AE exhibited a unique carnitine metabolism signature. Longitudinal follow‐up identified seven metabolites significantly associated with clinical remission in patients with AE.

**Conclusion:**

This study systematically maps shared and distinct metabolic perturbations in different types of encephalitis and suggests potential metabolic features associated with AE subtype‐specific pathophysiology, providing a preliminary metabolomic basis for precision diagnosis.

## Graphical abstract

Cerebrospinal fluid metabolomics in 232 patients reveals shared and distinct metabolic disruptions across encephalitis types. Diagnostic models for viral encephalitis were developed (AUC > 0.8). Subgroup analysis of autoimmune encephalitis indicated a unique metabolic signature in GFAP antibody‐positive cases, and longitudinal analysis identified treatment‐response biomarkers.

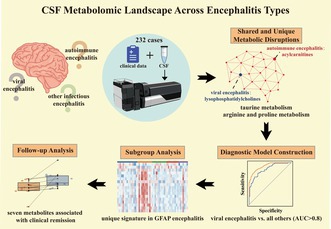

**Abbreviations**
AE: autoimmune encephalitis
AUC: area under the receiver operating characteristic curve
CI: confidence interval
CSF: cerebrospinal fluid
FDR: false discovery rate
GFAP: glial fibrillary acidic protein
HMDB: Human Metabolome Database
LGI‐1: leucine‐rich glioma‐inactivated 1
MOG: myelin oligodendrocyte glycoprotein
NE: non‐encephalitis disease control
NMDAR: N‐methyl‐D‐aspartate receptor
OIE: other infectious encephalitis
OPLS‐DA: orthogonal partial least‐squares discriminant analysis
PCA: principal component analysis
ROC: receiver operating characteristic
VE: viral encephalitis
VIP: variable importance in projection value
WBC: white blood cell

## 1 Introduction

Encephalitis, a neurological inflammatory condition, presents a significant global health burden and poses considerable diagnostic challenges in clinical practice [1, 2]. This disease has a diverse and complex etiology, primarily encompassing viral encephalitis (VE) caused by direct viral invasion and autoimmune encephalitis (AE), which results from immune‐mediated attacks on the nervous system [1]. Despite differing underlying mechanisms, various types of encephalitis frequently present with overlapping and non‐specific clinical features. Current diagnostics rely on detecting autoantibodies or pathogens, and approximately 50% of patients lack a definitive etiology, underscoring the urgent need to understand the molecular underpinnings of encephalitis and discover novel biomarkers [3].

In recent years, several studies conducted at the molecular level have progressively revealed shared neuroinflammatory pathways and significant etiological heterogeneity in the pathogenesis of various types of encephalitis [4, 5, 6, 7, 8]. Against this backdrop, cerebrospinal fluid (CSF) metabolomics has garnered widespread attention due to its ability to directly reflect the real‐time pathophysiological state and metabolic activity of the central nervous system [9, 10]. CSF offers a unique window into neuroinflammation‐related metabolic disturbances, providing clues for disease mechanisms and diagnostic markers [11].

Existing CSF metabolomic studies on encephalitis have largely followed conventional approaches, focusing primarily on metabolite dysregulation and pathways previously reported in other neurological diseases, with targeted detection and validation in small cohorts [12, 13, 14]. Accumulating evidence now suggests that multiple pathways (e.g., tryptophan–kynurenine and nitric oxide synthesis) may form the common metabolic foundation of CNS inflammation [12]. However, an understanding of the etiological heterogeneity of encephalitis remains relatively limited. To date, only a few metabolites have been identified as potential biomarkers for discriminating AE from VE, including N‐methyl‐d‐aspartate receptor (NMDAR) antibody encephalitis and multiple sclerosis, CSF kynurenine levels and their ratio to tryptophan, isobutyryl‐L‐carnitine and isovalerylcarnitine, as well as certain lipid molecules [14, 15]. Furthermore, the metabolic underpinnings of AE subtypes remain inadequately investigated, despite proteomic evidence suggesting that they may exhibit distinct molecular mechanisms [16].

In this regard, emerging untargeted metabolomic technologies offer unique methodological advantages over the current hypothesis‐driven targeted research paradigm in transcending established metabolic pathways [17]. This approach, which does not require pre‐specified target metabolites, enables global unbiased detection of metabolites in samples, thereby systematically characterizing comprehensive metabolic perturbation networks under disease conditions and overcoming the constraints of previous traditional targeted studies [17]. In recent years, small‐sample studies have successfully employed untargeted metabolomic strategies to identify key disease‐specific metabolites and associated pathways in specific encephalitis types, such as tick‐borne encephalitis [18]. These pioneering efforts demonstrate the utility of untargeted metabolomics for investigating the metabolic features of different encephalitis types.

Therefore, we aimed to systematically analyze the CSF metabolic profiles of different encephalitis types and conduct an in‐depth subtype analysis and longitudinal follow‐up of AE. We identified shared and distinct metabolic perturbation patterns across various encephalitis categories, constructed a systematic metabolic atlas for encephalitis, and developed the corresponding potential differential diagnostic models. These findings provide a new basis for characterizing the metabolic alterations associated with encephalitis and may contribute to advancing its precise clinical diagnosis and treatment.

## 2 Methods

### 2.1 Study Participants and Sample Collection

Patients with suspected encephalitis were prospectively enrolled at Xuanwu Hospital (2021–2024). Diagnoses were confirmed by two independent neurologists using internationally accepted criteria [19, 20, 21]. CSF samples were obtained via first lumbar puncture after admission, processed, and stored at −80°C. After exclusion, 232 participants were included in the study, including 10 with follow‐up CSF samples. Detailed inclusion and exclusion criteria are provided in Appendix S1.

### 2.2 Metabolites Extraction and Untargeted Metabolomics Analysis

Thawed CSF (100 μL) was mixed with 300 μL of protein‐precipitation solution (methanol–acetonitrile 2:1 v/v with 4 μg mL^−1^ internal standards), vortexed (1 min), sonicated (10 min in ice‐water bath), and incubated at −40°C for 2 h to extract metabolites. After centrifugation (13,000 rpm; 20 min; 4°C), 150 μL of supernatant was filtered (0.22 μm) for UPLC–MS/MS analysis.

Untargeted profiling was performed using an ACQUITY UPLC I‐Class Plus coupled to a Thermo QE mass spectrometer (Waters, USA) in positive and negative ion modes. Quality control samples (pooled aliquots of all CSF samples) were analyzed every 10 runs to monitor instrument stability. The detailed instrumental parameters are provided in Appendix S1.

### 2.3 Data Analysis and Metabolite Identification

Raw data were processed using Progenesis QI v3.0 (Nonlinear Dynamics, Newcastle, UK) for baseline filtering, peak identification, integration, retention time correction, alignment, and normalization. Metabolite identification was performed using HMDB, LipidMaps, METLIN, and a local database (mass tolerance 5–20 ppm). Metabolites were screened using a Compound Identification Scoring Tool [22]; those with a score < 36 (maximum 80) were excluded from the analysis. High‐confidence identifications required retention time match within ±0.3 min and fragmentation score > 0. Human endogenous metabolites were annotated using HMDB and CHEBI. The data were filtered by relative standard deviation (> 0.3 discarded), and missing values (> 50% per group) were removed, the remaining missing values were imputed with half the minimum intensity, and the data were log2‐transformed.

### 2.4 Statistical Analysis

All statistical analyses were performed using GraphPad Prism 10.6, R (4.4.1/4.2.2), and SPSS 27.0. Demographic characteristics were compared between the groups using the Kruskal–Wallis test (with Bonferroni correction) and *χ* ^2^ test. Metabolite data normality and variance homogeneity were assessed using Shapiro–Wilk and Levene's tests, respectively. Intergroup differences were evaluated using the two‐sided Wilcoxon rank‐sum test with Benjamini–Hochberg false discovery rate (FDR) correction. Differential metabolites were identified using orthogonal partial least‐squares discriminant analysis (OPLS‐DA) with permutation testing (variable importance in projection, VIP > 1, *p* < 0.05, FDR < 0.2). The correlation between all high‐confidence differential metabolites and CSF white blood cells (WBC) was analyzed using Spearman's correlation analysis. Additionally, all high‐confidence differential metabolites were analyzed using Kyoto Encyclopedia of Genes and Genomes (KEGG) pathway enrichment, and weighted metabolite correlation network analysis. The detailed network procedures are provided in Appendix S1.

Five LASSO‐logistic regression models were constructed to address the clinical diagnostic needs: (i) VE vs. AE, (ii) VE vs. non‐encephalitis control (NE), (iii) AE vs. NE, (iv) VE vs. all others, and (v) AE vs. all others. The participants were randomly assigned to the training or validation sets in a 7:3 ratio stratified by diagnosis. High‐confidence endogenous metabolites in the training set were standardized via z‐score normalization, and the same parameters were applied to the validation set. After independent *t*‐test screening, the top 40 metabolites (by *p*‐value) were selected for LASSO regression analysis. The model used L1 regularization (*α* = 1), with *λ* optimized via 5‐fold cross‐validation. A standard logistic regression model was refitted from the training set and validated. Receiver operating characteristic (ROC) curves were plotted using the pROC package; under the receiver operating characteristic curve > (AUC) with 95% confidence interval [CI] estimated using DeLong's method. The optimal cut‐off value was determined using Youden's index. Model performance was evaluated based on accuracy, sensitivity, specificity, positive likelihood ratio and negative likelihood ratio.

Subtype comparisons for AE (sample size > 6 per subtype) were performed using the Kruskal–Wallis test followed by FDR adjustment, with statistical significance defined as *p* < 0.05 and FDR < 0.2. Longitudinal metabolite changes between the acute and remission phases were assessed using the Wilcoxon signed‐rank test, with *p* < 0.05 considered significant.

## 3 Results

### 3.1 Participant Characteristics

**FIGURE 1:**
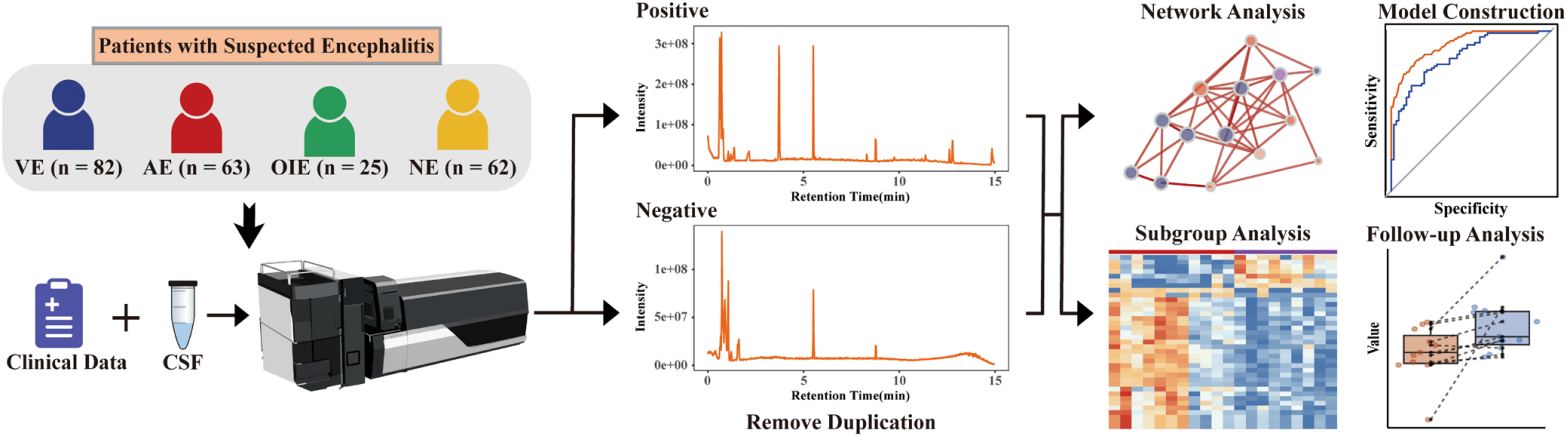
Flow chart of the present study. AE, autoimmune encephalitis; CSF, cerebrospinal fluid; NE, non‐encephalitis disease control; OIE, other infectious encephalitis; VE, viral encephalitis.

In total, 232 participants were enrolled in the study, including 82 diagnosed with VE, 63 with AE, 25 with other infectious encephalitis (OIE), and 62 with NE (Figure 1). The median patient ages (interquartile range) in the VE, AE, OIE, and NE groups were 35.0 (27.8–50.0), 47.0 (30.0–62.0), 46.0 (36.0–60.0), and 54.0 (37.8–62.3) years, respectively. Post hoc comparisons showed that the VE group was significantly younger than the AE (adjusted *p* = 0.031) and NE (adjusted *p* < 0.001) groups. No statistically significant differences in age were observed in the other pairwise comparisons. Furthermore, no significant difference was observed in the sex distribution among the four groups (*p* = 0.805). Detailed demographic characteristics and specific etiologies of each group are presented in Appendix S2: Table S1.

### 3.2 Comparison of CSF Metabolome Between Groups

**FIGURE 2:**
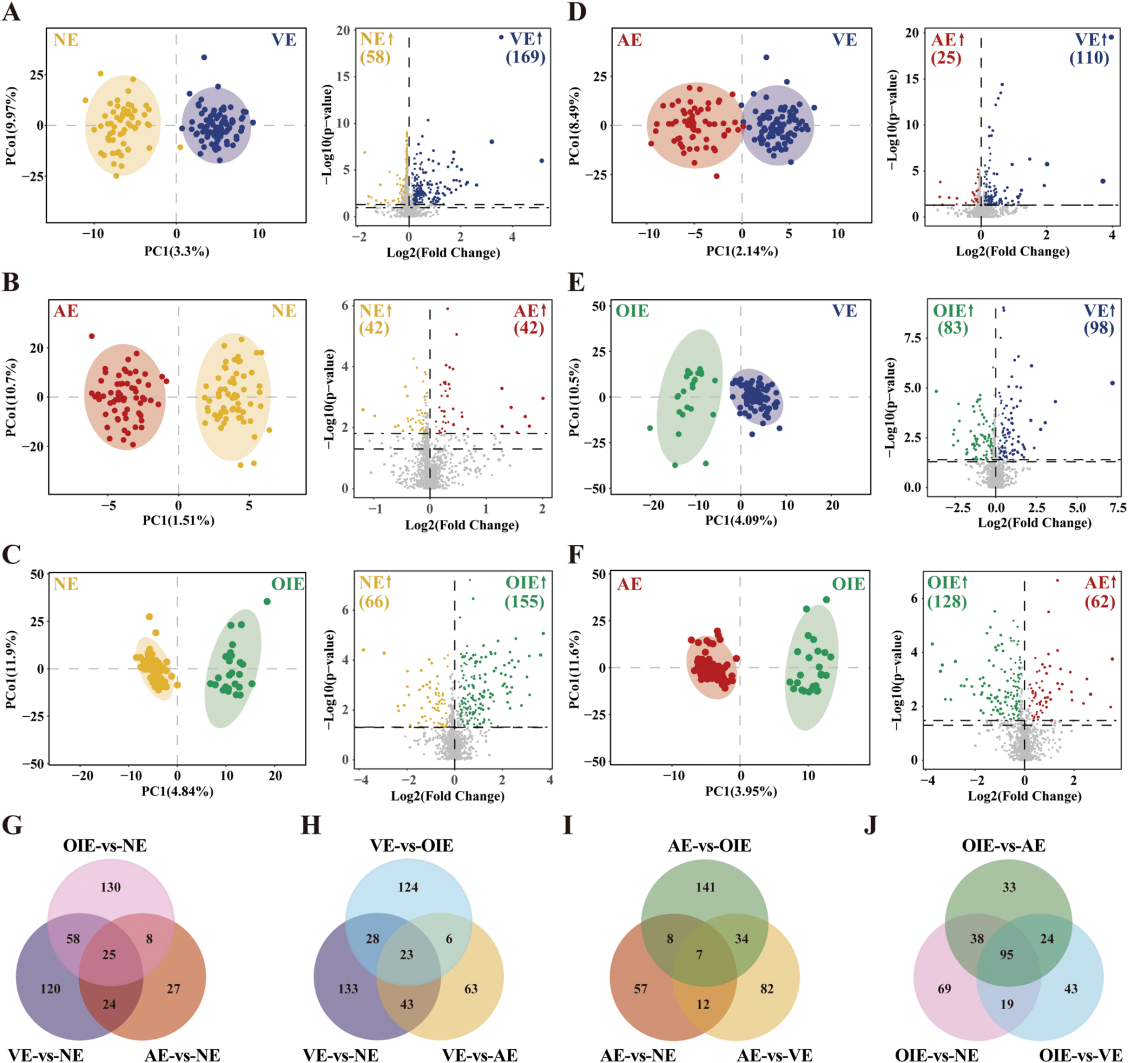
OPLS‐DA score plots, volcano plots, and Venn diagrams for inter‐group comparisons. Panels A–F show OPLS‐DA score plots and volcano plots for the pairwise comparisons of (A) VE vs. NE, (B) AE vs. NE, (C) OIE vs. NE, (D) VE vs. AE, (E) VE vs. OIE, and (F) AE vs. OIE. In the volcano plots, features with a VIP > 1, a *p*‐value < 0.05, and a FDR < 0.2 (two‐sided Benjamini–Hochberg test) were considered statistically significant; the number of such features for each comparison is indicated in parentheses. A dashed line represents *p* = 0.05, and a dash‐dot line represents FDR = 0.2. Panels G–J are Venn diagrams summarizing the distribution of differential metabolites across comparisons: (G) involving the NE group against all other groups; (H) involving the VE group against all other groups; (I) involving the AE group against all other groups; and (J) involving the OIE group against all other groups. AE, autoimmune encephalitis; NE, non‐encephalitis disease control; OIE, other infectious encephalitis; OPLS‐DA, orthogonal partial least squares‐discriminant analysis; VE, viral encephalitis; VIP, variable importance in projection value; FDR, false discovery rate.

In total, 120 of the 1532 compounds identified in the CSF of all participants were high‐confidence human endogenous metabolites (Appendix S2: Table S2). We conducted pairwise metabolomic comparisons among the VE, AE, OIE, and NE groups to characterize metabolic reprogramming features in the CSF across different encephalitis subtypes. The OPLS‐DA models demonstrated a clear separation between the pairs of groups. These models were validated by permutation tests, which confirmed the absence of overfitting, indicating distinct metabolic profile alterations among the encephalitis subtypes (Figure 2A–F, Appendix S1: Figure S1). In total, 489 differential metabolites were identified using univariate analysis in at least one pairwise comparison (Appendix S2: Table S2). These included 392 metabolites that differed between the encephalitis groups (VE, AE, or OIE) and the NE group (Figure 2G), 420 that involved VE (Figure 2H), 341 that involved AE (Figure 2I), and 321 that involved OIE (Figure 2J).

### 3.3 Clinical Associations and Pathway Enrichment of Differential Metabolites

**FIGURE 3:**
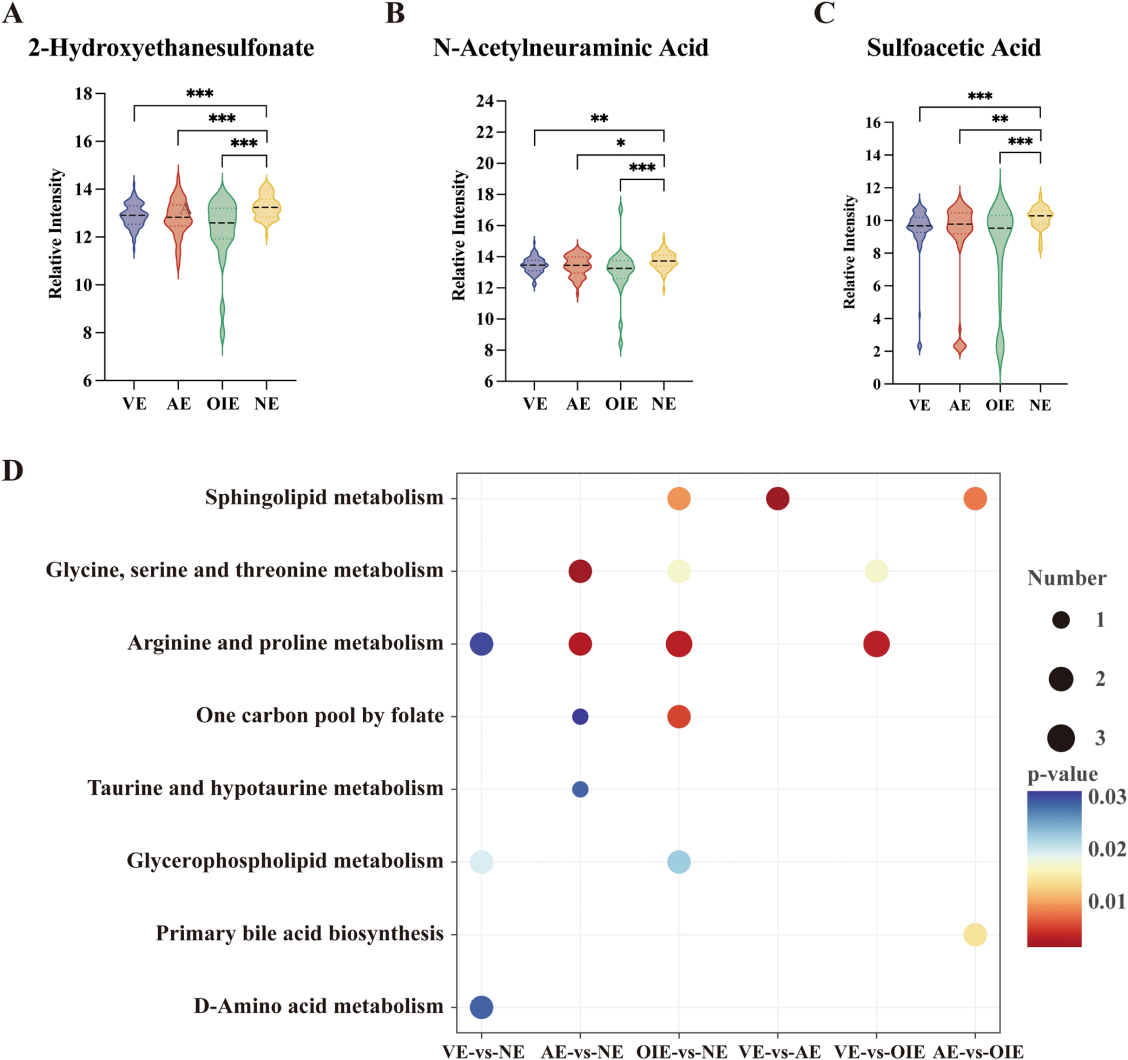
Alterations in important differential metabolites and perturbed pathways in different types of encephalitis. (A–C) Levels of two important differential metabolites (2‐hydroxyethanesulfonate and N‐acetylneuraminic acid) and a microbiota‐associated metabolite (sulfoacetic acid) across groups. *p‐value < 0.05; **p‐value < 0.01; ***p‐value < 0.001. (D) KEGG pathway enrichment analysis of important differential metabolites from pairwise comparisons. AE, autoimmune encephalitis; KEGG, Kyoto Encyclopedia of Genes and Genomes; NE, non‐encephalitis disease control; OIE, other infectious encephalitis; VE, viral encephalitis.

A total of 73 high‐confidence human endogenous metabolites were identified as important differential metabolites in at least one pairwise group comparison and selected for further investigation (Appendix S2: Table S2). Compared to the NE group, the VE, AE, and OIE groups had 37, 6, and 47 important differential metabolites, respectively. Among them, 2‐hydroxyethanesulfonate and N‐acetylneuraminic acid were consistently altered across all three encephalitis groups (Figure 3A,B). Additionally, sulfoacetic acid, which is likely of intestinal microbial origin, although it is not currently classified as endogenous, was downregulated in all encephalitis groups (Figure 3C). Pearson correlation analysis showed that 5‐hydroxy‐L‐tryptophan had the strongest positive correlation with CSF WBC count (*r* = 0.64, FDR < 0.001); all other metabolite correlations were < |*r*| = 0.6 (Appendix S2: Table S3). KEGG enrichment analysis indicated that important differential metabolites from the comparison between each encephalitis type and NE were significantly enriched in the arginine and proline metabolism pathway (VE‐NE: *p* = 0.030; AE‐NE: *p* = 0.003; OIE‐NE: *p* = 0.003; Figure 3D).

### 3.4 Alterations in Metabolic Network Structure Under Disease Conditions

**FIGURE 4:**
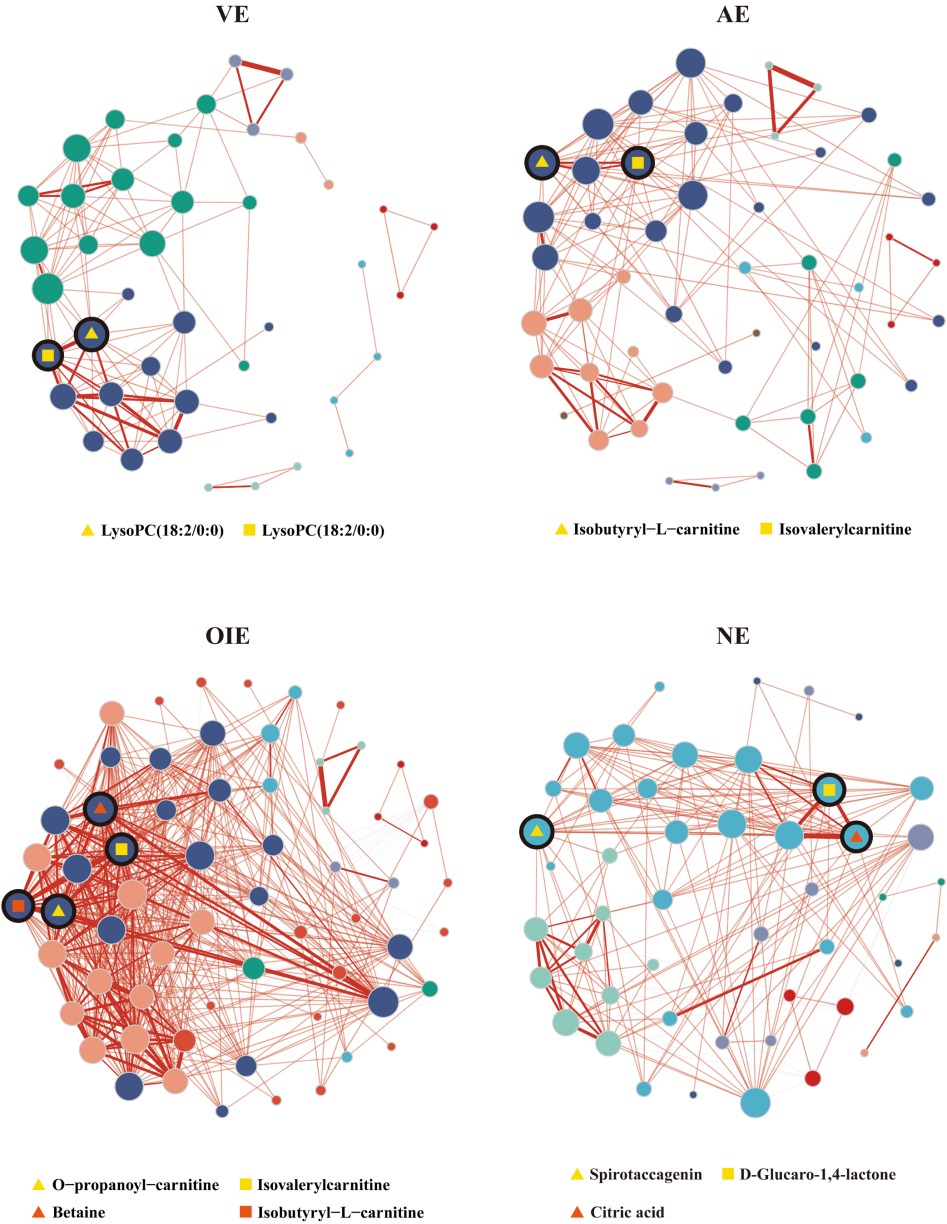
Metabolite correlation networks across different groups. Only correlations with a correlation coefficient > 0.6 and False Discovery Rate (FDR) < 0.05 were included in network construction and visualization. In each network, node size corresponds to degree centrality; node color indicates module assignment; edges represent positive (red) or negative (blue) correlations, with thickness and transparency scaled according to the magnitude of the correlation coefficient. Only key nodes are labeled with metabolite names.

We constructed metabolite correlation networks for each group based on the union of important differential metabolites. Global topological analysis revealed distinct structural differences between groups (Figure 4). Notably, the VE and AE groups exhibited well‐defined, highly modular network architectures, with whole network modularity coefficients of 0.48 and 0.46, respectively. This suggests that the metabolic perturbations in these disease states are organized into multiple functionally independent modules. Additionally, the VE (0.05) and AE (0.06) networks showed comparatively low edge density values, indicating relatively limited yet specific strong associations among the metabolites under these conditions. Furthermore, 100% of the statistically significant edges in the VE and AE networks were positively correlated, reflecting a high degree of global coordination in metabolic alterations, which is potentially indicative of a dominant unidirectional metabolic pattern in these conditions. However, the key nodes differed between the two networks: the VE network was centered on lysophosphatidylcholines (lysoPC (0:0/18:2) and lysoPC (18:2/0:0)), whereas the AE network was anchored by acylcarnitines (isobutyryl‐L‐carnitine and isovalerylcarnitine).

In contrast, the OIE network displayed high edge density (0.20) and natural connectivity (0.21), but low modularity (0.10), reflecting widespread, less structured metabolic associations. Meanwhile, the NE group exhibited intermediate modularity (0.34), with key nodes, including spirotaccagenin, D‐glucaro‐1,4‐lactone, and citric acid, linked to lipid detoxification and basal energy metabolism, respectively. The detailed topological metrics are listed in Table 1.

**TABLE 1:** Topological attributes in metabolic networks across different groups.

	VE	AE	OIE	NE
*LCC*				
Relative LCC size	0.44	0.55	0.78	0.55
Clustering coefficient	0.73	0.71	0.71	0.68
Modularity	0.41	0.39	0.08	0.31
Positive edge percentage	100	100	94.7	93.4
Edge density	0.24	0.18	0.33	0.25
Natural connectivity	0.15	0.11	0.28	0.15
*Whole network*				
Clustering coefficient	0.73	0.76	0.74	0.63
Modularity	0.48	0.46	0.10	0.34
Positive edge percentage	100	100	94.8	93.6
Edge density	0.05	0.06	0.20	0.08
Natural connectivity	0.05	0.05	0.21	0.07
Key nodes	LysoPC(0:0/18:2) LysoPC(18:2/0:0)	Isobutyryl‐L‐carnitine Isovalerylcarnitine	O‐propanoyl‐carnitine Betaine Isobutyryl‐L‐carnitine Isovalerylcarnitine	Spirotaccagenin D‐Glucaro‐1,4‐lactone Citric acid

Abbreviations: AE, autoimmune encephalitis; LCC, Largest connected component; NE, non‐encephalitis; OIE, other infectious encephalitis; VE, viral encephalitis.

### 3.5 Screening of Candidate Diagnostic Metabolites and Construction of a Discriminant Model

**FIGURE 5:**
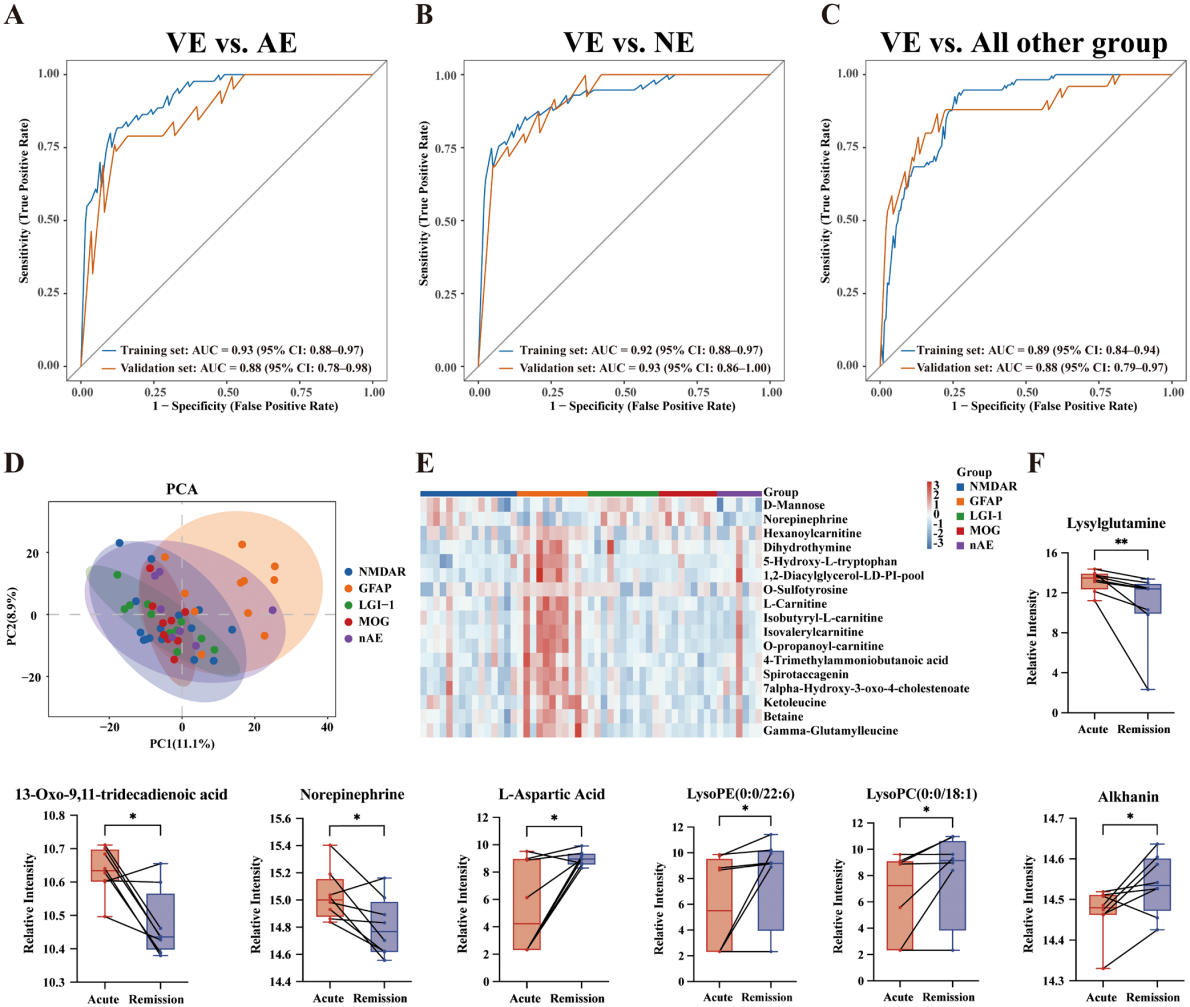
Diagnostic, subtype‐specific, and longitudinal metabolic findings in encephalitis. (A–C) Receiver operating characteristic curves and the corresponding areas under the curve for Model I, Model II and Model III. (D) PCA score plot showing the separation trend among AE subtypes. (E) Heatmap of 17 important differential metabolites across AE subtypes. (F) Box plots showing metabolite levels that significantly decreased or increased in CSF during the remission phase compared to the acute phase in a follow‐up cohort. *p < 0.05; **p < 0.01. AE, autoimmune encephalitis; CSF, cerebrospinal fluid; GFAP, glial fibrillary acidic protein antibody‐positive; LGI‐1, leucine‐rich glioma‐inactivated 1 antibody‐positive; MOG, myelin oligodendrocyte glycoprotein antibody‐positive; nAE, seronegative autoimmune encephalitis; NE, non‐encephalitis disease control; NMDAR, N‐methyl‐d‐aspartate receptor antibody‐positive; PCA, principal component analysis; VE, viral encephalitis.

Five diagnostic models were developed to address distinct clinical diagnostic needs. Among them, three models designed to differentiate VE from other encephalitis types demonstrated robust discriminative performance. Conversely, the models distinguishing AE from NE and AE from all other groups (VE, OIE, and NE) performed suboptimally. Model I included eight features to discriminate VE from AE and achieved an AUC of 0.93 (95% CI: 0.88–0.97) in the training set and 0.88 (95% CI: 0.78–0.98) in the validation set (Figure 5A), with a sensitivity/specificity of 81.8%/89.5% and 78.9%/88.0%, respectively. Model II comprised nine features for distinguishing VE from NE and yielded an AUC of 0.92 (95% CI: 0.88–0.97) and 0.93 (95% CI: 0.86–1.00) (Figure 5B), and a sensitivity/specificity of 75.4%/95.3% and 68.0%/100% in the training and validation sets, respectively. Model III, which included 10 features to differentiate VE from all other groups (including AE, OIE, and NE), had an AUC of 0.89 (95% CI: 0.84–0.94) and 0.88 (95% CI: 0.79–0.97) (Figure 5C), and a sensitivity/specificity of 93.0%/74.3% and 88.0%/80.0% in the training and validation sets, respectively. The detailed panel compositions and performance metrics are provided in Appendix S2: Tables S4 and S5. These findings are at the discovery level and require targeted validation of key metabolites in independent cohorts.

### 3.6 Subtype Analysis of Autoimmune Encephalitis

Metabolomic profiling was conducted across AE subtypes comprising more than six cases, including NMDAR antibody‐positive (*n* = 15), glial fibrillary acidic protein (GFAP) antibody‐positive (*n* = 11), leucine‐rich glioma‐inactivated 1 (LGI‐1) antibody‐positive (*n* = 11), myelin oligodendrocyte glycoprotein (MOG) antibody‐positive (*n* = 9), and seronegative AE (*n* = 7). Principal component analysis (PCA) revealed a separation trend among the subtypes, with GFAP‐positive cases showing the clearest distinction from other subtypes (Figure 5D). Kruskal–Wallis analysis identified 71 metabolites that differed significantly across the subtypes (*p* < 0.05, FDR < 0.2). Hierarchical clustering of the 17 important differential metabolites highlighted pronounced changes in the GFAP‐positive cases (Figure 5E), indicating subtype‐specific metabolic reprogramming. Notably, six of the GFAP‐positive‐specific metabolites—hexanoylcarnitine, L‐carnitine, isobutyryl‐L‐carnitine, 4‐trimethylammoniobutanoic acid, isovalerylcarnitine, and O‐propanoyl‐carnitine—belong to the carnitine metabolism pathway.

### 3.7 Follow‐Up Analysis of Autoimmune Encephalitis

A second CSF sample was obtained from 10 participants in the cohort, eight of whom were diagnosed with AE and included in the follow‐up analysis. The AE subgroup consisted of NMDAR‐ (*n* = 4), MOG‐ (*n* = 2), GFAP‐ (*n* = 1), and LGI‐1‐antibody‐positive (*n* = 1) cases. All patients showed significant clinical remission at follow‐up. Among these patients, longitudinal metabolomic analysis identified seven important differential metabolites that changed significantly between the acute and remission phases. Specifically, three differential metabolites were downregulated (lysylglutamine, *p* = 0.007; 13‐oxo‐9,11‐tridecadienoic acid, *p* = 0.023; norepinephrine, *p* = 0.039) and four were upregulated (L‐aspartic acid, *p* = 0.023; LysoPE(0:0/22:6), *p* = 0.036; LysoPC(0:0/18:1), *p* = 0.036; alkhanin, *p* = 0.039) (Figure 5F). Notably, 13‐oxo‐9,11‐tridecadienoic acid, LysoPE(0:0/22:6), and LysoPC(0:0/18:1) showed consistent directional changes in two additional longitudinally sampled patients diagnosed with syphilitic or viral infections.

## 4 Discussion

In this study, we used untargeted metabolomics to systematically characterize the metabolic profiles of clinically similar diseases VE, AE, and OIE, and identify differential and shared metabolic features, as well as the core regulatory pathways between VE and AE. Subsequently, we leveraged these metabolomic characteristics and developed a metabolite‐based diagnostic model to distinguish VE from other encephalitis types. Additional investigations revealed metabolic heterogeneity in GFAP antibody‐positive AE compared to other AE subtypes, and longitudinal analysis tracked dynamic metabolite changes during the transition from acute to remission phases in patients with AE.

Previous studies on encephalitis metabolism have focused on classical pathways such as tryptophan‐kynurenine and nitric oxide synthesis. In contrast, this study highlighted two previously underexplored metabolic pathways, taurine and arginine–proline metabolism, that were dysregulated across encephalitis types. We observed consistent downregulation of two taurine‐derived metabolites—2‐hydroxyethanesulfonate and sulfoacetic acid across all three encephalitis groups compared to the NE controls. Both metabolites are reportedly produced in the human body via gut microbial metabolism of taurine, although their definitive synthesis pathways within human cells remain unclear [23, 24]. While previous studies have reported inconsistent changes in taurine in the CSF of patients with encephalitis, we observed consistent alterations in its downstream metabolites [25, 26, 27, 28]. This suggests that disruption of the taurine metabolic network, whether driven by host or microbial processes, is a common metabolic hallmark of encephalitis. KEGG enrichment analysis further confirmed that the arginine‐proline metabolism pathway was commonly dysregulated across all encephalitis groups, suggesting its involvement in amino acid reprogramming and immune regulation.

Additionally, N‐acetylneuraminic acid was consistently downregulated in all three encephalitis groups. As sialic acid is widely present in human cells, particularly in the brain, and is often positioned terminally on gangliosides and polysialic acids, N‐acetylneuraminic acid participates in various physiological processes such as cell adhesion, signaling, and immune modulation by regulating molecular interactions and brain plasticity [29]. Notably, the NE control group included patients with neurological diseases, such as acute cerebral infarction and tumors, which may elevate free N‐acetylneuraminic acid [30, 31, 32, 33, 34, 35]. Therefore, the decrease in CSF N‐acetylneuraminic acid in encephalitis may reflect relatively milder and potentially reversible brain injury. In addition, one patient with *Streptococcus pneumoniae* meningitis exhibited a significantly higher N‐acetylneuraminic acid level than all other participants, with a CSF metagenomic next‐generation sequencing pathogen sequence count of approximately 10,000. This case suggests that significant N‐acetylneuraminic acid elevation might occur during specific stages of extremely high bacterial load proliferation, consistent with previous reports of bacterial meningitis, but not necessarily extending to encephalitis types dominated by viral or autoimmune damage [33, 35, 36].

Disruption of cell membrane integrity and mitochondrial dysfunction are two core pathological processes that drive neuronal damage in VE and AE and may induce characteristic metabolic disturbances [37, 38, 39]. However, existing single‐metabolite analyses are unable to reveal the global architecture of metabolic reprogramming under these conditions. In contrast, the topological network analysis in this study revealed that the VE and AE groups exhibited highly similar network characteristics, high modularity, and high positive synergy, which were markedly different from those observed in the OIE and NE groups. This suggests that despite differing initiating factors, these two pathological processes may drive the metabolic system away from a homeostatic state rich in negative feedback regulation toward a highly synergistic “disease state” dominated by intense pathological processes. Further analyses revealed distinct core regulatory hubs within this common framework. The VE network was centered on lysophosphatidylcholine, aligning with the pathological focus on virus‐triggered membrane phospholipid degradation, phospholipase activation, and subsequent lipid‐mediated inflammatory cascades [14, 40]. In contrast, the core hub of the AE network was acylcarnitine, indicating a mitochondrial energy metabolism crisis in neurons and glial cells under immune attack [41, 42, 43].

Moreover, the findings demonstrate that the same set of differential metabolites could form regulatory networks with drastically different topological structures in distinct disease contexts. This finding indicates that the functional importance of a metabolite depends critically on its systemic regulatory role in addition to its absolute abundance. A key example is acylcarnitine; although its relative level was higher in the VE group than that in the AE group, it emerged as a highly connected and central core hub only in the AE metabolic network. Meanwhile, the core hub in the VE network was occupied by lysophosphatidylcholine. These findings highlight the advantages of multi‐metabolite approaches. Accordingly, we used untargeted metabolomics and machine learning and developed diagnostic models with the potential to distinguish VE from other encephalitis types at the discovery level. However, models differentiating AE from NE or other groups were not effective, likely because of high within‐group heterogeneity.

Therefore, we explored the metabolic heterogeneity of AE. Analysis across AE subtypes revealed that GFAP antibody‐positive encephalitis exhibits a distinct metabolic signature, characterized mainly by the specific upregulation of acylcarnitines and related metabolites. GFAP encephalitis is widely believed to be primarily mediated by GFAP‐specific CD8^+^ T cells; however, its associated metabolic alterations remain unknown [44]. Based on the present results, we speculate that immune attacks targeting astrocytes may specifically impair the mitochondrial capacity to process branched‐chain amino acids and certain lipids. Acylcarnitines, which are esters formed by conjugating fatty acids or specific amino acids with L‐carnitine, play key roles in cellular energy metabolism [45, 46]. When mitochondrial or peroxisomal dysfunction impedes fatty acid β‐oxidation or amino acid degradation, intermediate metabolite acylcarnitines accumulate intracellularly and are effluxed via specific transporters, elevating extracellular levels [46]. In response, cells may activate a compensatory program centered on the carnitine system to counteract this “metabolic blockade,” by upregulating L‐carnitine and its synthetic precursor 4‐trimethylammoniobutanoic acid to conjugate with accumulated incompletely metabolized acyl groups [45, 46]. Subsequently, these acylcarnitines are transported out of the mitochondria, alleviating the cytotoxicity from toxic acyl group accumulation. This unique metabolic phenotype provides a new molecular perspective for understanding the distinct pathology of GFAP encephalitis and suggests that future research could focus on modulating branched‐chain amino acid metabolic flux or enhancing cellular metabolic buffering capacity, offering new avenues for potential therapeutic interventions.

Subsequently, longitudinal metabolomic analysis of eight patients with AE revealed characteristic metabolic changes during remission compared to those in the acute phase, suggesting a systemic shift in the central nervous system from a state of neuroinflammatory attack to active repair and reconstruction. Specifically, the membrane phospholipid precursors LysoPC(0:0/18:1) and LysoPE(0:0/22:6) were significantly upregulated during remission, providing essential materials for synaptic and membrane remodeling, and collectively indicating enhanced anabolism and membrane stability [47]. L‐aspartate was downregulated acutely, likely due to increased metabolic consumption, whereas the stress markers lysylglutamine and norepinephrine, which were elevated in the acute phase, normalized during remission, reflecting diminished inflammation and a moderate hyperimmune state [48]. Notably, previously identified differential metabolites related to taurine, arginine/proline, and carnitine metabolism did not change significantly during clinical remission. This does not negate their importance in AE disease processes, but may reflect the inherent complexity of the disease: underlying metabolic disturbances and immune dysregulation can persist even during clinical remission, potentially contributing to relapse and complicating long‐term management.

Despite the positive outcomes, this study had some limitations. First, age differences across encephalitis groups were considered inherent clinical features rather than confounders; therefore, age correction was not applied to avoid masking genuine metabolic signals. Second, the diagnostic models were trained and tested using only a single internal dataset, and no independent external validation cohorts were available. Thus, the reported AUC values may be optimistic, and their generalizability to other populations requires further verification. Moreover, key metabolic signatures identified in this untargeted discovery study require targeted quantitative validation in independent cohorts. Third, the OIE group included only 25 patients, which was relatively small compared with the other groups. This disparity may have affected the statistical robustness of the analyses involving OIE, and these findings should be interpreted with caution. Consequently, larger independent cohorts, particularly those with more patients with OIE, are required for validation.

## 5 Conclusion

In this study, we used untargeted metabolomics to systematically map the metabolic profiles of VE, AE, and OIE. The findings confirmed that the taurine and arginine‐proline pathways are commonly dysregulated across encephalitis types and indicated that VE and AE share a highly synergistic‐modular metabolic signature, despite differing etiologies. The key metabolites, lysophosphatidylcholine in VE and acylcarnitines in AE, correspond to the two major pathological paradigms: membrane phospholipid degradation and mitochondrial energy crisis. Diagnostic models constructed based on these metabolic signatures demonstrated the potential to distinguish VE from other conditions during internal validation. Furthermore, this study showed that GFAP antibody‐positive encephalitis exhibits a metabolic signature marked by acylcarnitine accumulation. In summary, these findings provide a foundation for characterizing the metabolic alterations associated with core disease processes and developing differential clinical diagnostic models.

## Author Contributions

Conceptualization: Y.C., J.Z., and L.W.; Data curation: Y.C., H.N., Z.W., D.G., H.Y., Y.Z., J.L., L.W., and W.C.; Investigation: Y.C., J.Z., and L.W.; Methodology: Y.C., J.Z., and L.W.; Resources: L.W. and W.C.; Visualization: Y.C.; Writing – original draft: Y.C. and J.Z.; Writing – review and editing: L.W. All the authors have read and agreed to the published version of the manuscript.

## Funding

This work was supported by grants from the Ministry of Science and Technology of the People's Republic of China (MOST) (key project no. 2020YFC2005403) and “Yangfan 3.0” Diagnostic and Therapeutic Capability Enhancement Project of Beijing Municipal Hospital Administration (ZLRK202515).

## Ethics Statement

This study was conducted in accordance with the Declaration of Helsinki and approved by the Institutional Ethical Standards Committee of Xuanwu Hospital, Capital Medical University (number: [2020]104). Written consent for the study was obtained from the families and patient representatives.

## Consent

The authors have nothing to report.

## Conflicts of Interest

The authors declare no conflicts of interest.

## Supporting information


**Figure S1:** Permutation test results (200 permutations) for the orthogonal partial least squares‐discriminant analysis (OPLS‐DA) models.

**Table S1:** Demographic characteristics and etiologies by patient group.
**Table S2:** Metabolite identification and differential analysis results.
**Table S3:** The Correlation between important differential metabolites and white blood cells in cerebrospinal fluid.
**Table S4:** Logistic regression coefficients and odds ratios for differential diagnostic models.
**Table S5:** Performance metrics of the models.

## Data Availability

The datasets used and/or analyzed in the current study are available from the corresponding author upon reasonable request.

## References

[1] Y. Segal , O. Rotschild , Y. Mina , et al., “Epidemiology of Autoimmune Encephalitis and Comparison to Infectious Causes‐Experience From a Tertiary Center,” Annals of Clinical Translational Neurology 11, no. 9 (2024): 2337–2349. 39030965 10.1002/acn3.52147 PMC11537142

[2] J. D. Steinmetz , K. M. Seeher , N. Schiess , et al., “Global, Regional, and National Burden of Disorders Affecting the Nervous System, 1990–2021: A Systematic Analysis for the Global Burden of Disease Study 2021,” Lancet Neurology 23, no. 4 (2024): 344–381. 38493795 10.1016/S1474-4422(24)00038-3 PMC10949203

[3] N. M. Vora , R. C. Holman , J. M. Mehal , C. A. Steiner , J. Blanton , and J. Sejvar , “Burden of Encephalitis‐Associated Hospitalizations in the United States, 1998–2010,” Neurology 82, no. 5 (2014): 443–451. 24384647 10.1212/WNL.0000000000000086

[4] S. Fan , X. He , Z. Zhu , et al., “Integrating Host Transcriptomic Signatures for Distinguishing Autoimmune Encephalitis in Cerebrospinal Fluid by Metagenomic Sequencing,” Cell & Bioscience 13, no. 1 (2023): 111. 37332019 10.1186/s13578-023-01047-x PMC10278324

[5] X. Liu , K. Fan , Q. Lin , et al., “Serum‐Derived Exosomal miR‐140‐5p as a Promising Biomarker for Differential Diagnosis of Anti‐NMDAR Encephalitis With Viral Encephalitis,” Frontiers in Immunology 13 (2022): 840003. 35273615 10.3389/fimmu.2022.840003 PMC8902043

[6] J. Yan , K. Kothur , S. Mohammad , et al., “CSF Neopterin, Quinolinic Acid and Kynurenine/Tryptophan Ratio Are Biomarkers of Active Neuroinflammation,” eBioMedicine 91 (2023): 104589. 37119734 10.1016/j.ebiom.2023.104589 PMC10165192

[7] L. L. Xiong , L. L. Xue , Y. J. Chen , et al., “Proteomics Study on the Cerebrospinal Fluid of Patients With Encephalitis,” ACS Omega 6, no. 25 (2021): 16288–16296. 34235299 10.1021/acsomega.1c00367 PMC8246475

[8] B. D. Michael , M. J. Griffiths , J. Granerod , et al., “Characteristic Cytokine and Chemokine Profiles in Encephalitis of Infectious, Immune‐Mediated, and Unknown Aetiology,” PLoS One 11, no. 1 (2016): e0146288. 26808276 10.1371/journal.pone.0146288 PMC4726626

[9] O. N. Plaatjie , A. M. T. van Furth , M. van der Kuip , and S. Mason , “LC‐MS Metabolomics and Lipidomics in Cerebrospinal Fluid From Viral and Bacterial CNS Infections: A Review,” Frontiers in Neurology 15 (2024): 1403312. 39161867 10.3389/fneur.2024.1403312 PMC11330781

[10] D.‐K. Vo and K. T. L. Trinh , “Emerging Biomarkers in Metabolomics: Advancements in Precision Health and Disease Diagnosis,” International Journal of Molecular Sciences 25, no. 23 (2024): 13190. 39684900 10.3390/ijms252313190 PMC11642057

[11] D. S. Wishart , “Metabolomics for Investigating Physiological and Pathophysiological Processes,” Physiological Reviews 99, no. 4 (2019): 1819–1875. 31434538 10.1152/physrev.00035.2018

[12] J. Yan , U. Kuzhiumparambil , S. Bandodkar , R. C. Dale , and S. Fu , “Cerebrospinal Fluid Metabolomics: Detection of Neuroinflammation in Human Central Nervous System Disease,” Clinical & Translational Immunology 10, no. 8 (2021): e1318. 34386234 10.1002/cti2.1318 PMC8343457

[13] X. Li , X. Qin , Y. Xie , et al., “Metabolic Characterization of Cerebrospinal Fluid for Patients With Autoimmune Encephalitis: A Preliminary Study,” CNS Neuroscience & Therapeutics 31, no. 1 (2025): e70203. 39749658 10.1111/cns.70203 PMC11696248

[14] A. Al‐Mekhlafi , F. H. Waqas , M. Krueger , et al., “Elevated Phospholipids and Acylcarnitines C4 and C5 in Cerebrospinal Fluid Distinguish Viral CNS Infections From Autoimmune Neuroinflammation,” Journal of Translational Medicine 21, no. 1 (2023): 776. 37919735 10.1186/s12967-023-04637-y PMC10621113

[15] K. W. Sühs , N. Novoselova , M. Kuhn , et al., “Kynurenine Is a Cerebrospinal Fluid Biomarker for Bacterial and Viral Central Nervous System Infections,” Journal of Infectious Diseases 220, no. 1 (2019): 127–138. 30721966 10.1093/infdis/jiz048

[16] S. Räuber , C. B. Schroeter , C. Strippel , et al., “Cerebrospinal Fluid Proteomics Indicates Immune Dysregulation and Neuronal Dysfunction in Antibody Associated Autoimmune Encephalitis,” Journal of Autoimmunity 135 (2023): 102985. 36621173 10.1016/j.jaut.2022.102985

[17] A. Di Minno , M. Gelzo , M. Stornaiuolo , et al., “The Evolving Landscape of Untargeted Metabolomics,” Nutrition, Metabolism, and Cardiovascular Diseases 31, no. 6 (2021): 1645–1652. 10.1016/j.numecd.2021.01.008 33895079

[18] R. Liang , Y. Li , J. Li , et al., “Metabolomic Profiling of Cerebrospinal Fluid Reveals Metabolite Biomarkers in Tick‐Borne Encephalitis Patient,” Journal of Medical Virology 96, no. 11 (2024): e70082. 39569456 10.1002/jmv.70082 PMC11579828

[19] H. Abboud , J. C. Probasco , S. Irani , et al., “Autoimmune Encephalitis: Proposed Best Practice Recommendations for Diagnosis and Acute Management,” Journal of Neurology, Neurosurgery, and Psychiatry 92, no. 7 (2021): 757–768. 33649022 10.1136/jnnp-2020-325300 PMC8223680

[20] F. Graus , M. J. Titulaer , R. Balu , et al., “A Clinical Approach to Diagnosis of Autoimmune Encephalitis,” Lancet Neurology 15, no. 4 (2016): 391–404. 26906964 10.1016/S1474-4422(15)00401-9 PMC5066574

[21] A. Venkatesan , A. R. Tunkel , K. C. Bloch , et al., “Case Definitions, Diagnostic Algorithms, and Priorities in Encephalitis: Consensus Statement of the International Encephalitis Consortium,” Clinical Infectious Diseases 57, no. 8 (2013): 1114–1128. 23861361 10.1093/cid/cit458 PMC3783060

[22] L. W. Sumner , Z. Lei , B. J. Nikolau , et al., “Proposed Quantitative and Alphanumeric Metabolite Identification Metrics,” Metabolomics 10, no. 6 (2024): 1047–1049.

[23] X. Liu , Y. Wei , J. Zhang , Y. Zhou , Y. du , and Y. Zhang , “Isethionate Is an Intermediate in the Degradation of Sulfoacetate by the Human Gut Pathobiont *Bilophila wadsworthia* ,” Journal of Biological Chemistry 299, no. 8 (2023): 105010. 37414148 10.1016/j.jbc.2023.105010 PMC10413351

[24] J. H. Fellman , E. S. Roth , N. A. Avedovech , and K. D. Mccarthy , “The Metabolism of Taurine to Isethionate,” Archives of Biochemistry and Biophysics 204, no. 2 (1980): 560–567. 6255874 10.1016/0003-9861(80)90068-5

[25] S. L. Staal , S. E. Olie , M. van Weeghel , et al., “Cerebrospinal Fluid Metabolome in Central Nervous System Infections: A Study of Diagnostic Accuracy,” Annals of Neurology 98, no. 4 (2025): 851–863. 40525505 10.1002/ana.27291 PMC12542320

[26] J. Launes , J. Sirén , L. Viinikka , L. Hokkanen , and P. J. Lindsberg , “Does Glutamate Mediate Brain Damage in Acute Encephalitis?,” Neuroreport 9, no. 4 (1998): 577–581. 9559919 10.1097/00001756-199803090-00003

[27] C. Zhang , T. Zhou , S. Qiao , et al., “Taurine Attenuates Neuronal Ferroptosis by CSF‐Derived Exosomes of GABABR Encephalitis Through GABABR/NF2/P‐YAP Pathway,” Molecular Neurobiology 62, no. 7 (2025): 9052–9073. 40085353 10.1007/s12035-025-04819-3

[28] X. Song , Y. Wang , and K. Zheng , “Taurine Ameliorates Viral Encephalitis by Restoring PRKN‐Mediated Mitophagy,” Autophagy 21, no. 11 (2025): 2523–2525. 40702688 10.1080/15548627.2025.2538767 PMC12542610

[29] B. Wang , T. Zhang , S. Tang , C. Liu , C. Wang , and J. Bai , “The Physiological Characteristics and Applications of Sialic Acid,” NPJ Science of Food 9 (2025): 28. 40011515 10.1038/s41538-025-00390-2 PMC11865545

[30] M. Sindelar , J. P. Dyke , R. S. Deeb , et al., “Untargeted Metabolite Profiling of Cerebrospinal Fluid Uncovers Biomarkers for Severity of Late Infantile Neuronal Ceroid Lipofuscinosis (CLN2, Batten Disease),” Scientific Reports 8 (2018): 15229. 30323181 10.1038/s41598-018-33449-0 PMC6189193

[31] M. Heimerl , T. Gausepohl , J. H. Mueller , and M. Ricke‐Hoch , “Neuraminidases—Key Players in the Inflammatory Response After Pathophysiological Cardiac Stress and Potential New Therapeutic Targets in Cardiac Disease,” Biology 11, no. 8 (2022): 1229. 36009856 10.3390/biology11081229 PMC9405403

[32] V. N. Fedorov , A. G. Kochetov , O. V. Liang , and V. I. Skvortsova , “Clinical and Laboratory Assessment of Indicators of Oxidative Status in the Cerebrospinal Fluid of Patients With Ischemic Stroke,” Zhurnal Nevrologii i Psikhiatrii Imeni SS Korsakova 111, no. 8 Pt 2 (2011): 31–34. 22224242

[33] S. P. Kulkarni , C. R. Mallikarjuna , and D. S. J. Murthy , “Cerebrospinal Fluid Free Sialic Acid and Aspartate Transaminase Levels in Meningitis,” Indian Journal of Clinical Biochemistry 21, no. 1 (2006): 185–188. 23105596 10.1007/BF02913093 PMC3453762

[34] L. Krüger , K. Biskup , C. G. Schipke , et al., “The Cerebrospinal Fluid Free‐Glycans Hex1 and HexNAc1Hex1Neu5Ac1 as Potential Biomarkers of Alzheimer's Disease,” Biomolecules 14, no. 5 (2024): 512. 38785920 10.3390/biom14050512 PMC11117705

[35] A. Darbari , N. R. Bhandari , B. K. Agrawal , and S. A. Bhambal , “Cerebrospinal Fluid N‐Acetyl Neuraminic Acid Estimation for Early Diagnosis and Differentiation of Bacterial Meningitis,” Indian Pediatrics 28, no. 5 (1991): 513–519. 1752679

[36] J. K. Sharma , B. S. Gupta , and V. M. Bhandari , “Cerebrospinal Fluid Free N‐Acetyl Neuraminic Acid Levels—A Diagnostic and Prognostic Test in Meningitis,” Journal of the Association of Physicians of India 37, no. 12 (1989): 757–759. 2636579

[37] Y. Wang , J. Lu , A. F. Carisey , et al., “Innate Immune and Metabolic Signals Induce Mitochondria‐Dependent Membrane Lysis via Mitoxyperiosis,” Cell 188, no. 25 (2025): 7155–7174.e25. 41317732 10.1016/j.cell.2025.11.002 PMC12875127

[38] T. K. Makar , P. R. Guda , S. Ray , et al., “Immunomodulatory Therapy With Glatiramer Acetate Reduces Endoplasmic Reticulum Stress and Mitochondrial Dysfunction in Experimental Autoimmune Encephalomyelitis,” Scientific Reports 13 (2023): 5635. 37024509 10.1038/s41598-023-29852-x PMC10079956

[39] X. Song , Y. Wang , W. Zou , et al., “Inhibition of Mitophagy via the EIF2S1‐ATF4‐PRKN Pathway Contributes to Viral Encephalitis,” Journal of Advanced Research 73 (2025): 199–217. 39103048 10.1016/j.jare.2024.08.003 PMC12225914

[40] H. Liu , K. Qiu , Q. He , Q. Lei , and W. Lu , “Mechanisms of Blood‐Brain Barrier Disruption in Herpes Simplex Encephalitis,” Journal of Neuroimmune Pharmacology 14, no. 2 (2019): 157–172. 30456443 10.1007/s11481-018-9821-6

[41] J. Liu , Y. Huang , T. Qian , et al., “Exploring the Neuroprotective Role of Artesunate in Mouse Models of Anti‐NMDAR Encephalitis: Insights From Molecular Mechanisms and Transmission Electron Microscopy,” Cell Communication and Signaling: CCS 22, no. 1 (2024): 269. 38745240 10.1186/s12964-024-01652-4 PMC11094908

[42] L. Kong , X. Yang , A. Sun , X. Yang , X. Zhao , and S. Wang , “Rapamycin Alleviates Mitochondrial Dysfunction in Anti‐NMDAR Encephalitis Mice,” International Immunopharmacology 132 (2024): 111910. 38552295 10.1016/j.intimp.2024.111910

[43] C. Li , J. Y. Chen , Y. Peng , et al., “CSF Mitochondrial N‐Formyl Methionine Peptide as Complementary Diagnostic Tool in Anti‐NMDAR Encephalitis and Anti‐LGI1 Encephalitis,” Neuropsychiatric Disease and Treatment 20 (2024): 2629–2636. 39741905 10.2147/NDT.S482616 PMC11687102

[44] Y. Guo , V. Endmayr , A. Zekeridou , et al., “New Insights Into Neuropathology and Pathogenesis of Autoimmune Glial Fibrillary Acidic Protein Meningoencephalomyelitis,” Acta Neuropathologica 147, no. 1 (2024): 31. 38310187 10.1007/s00401-023-02678-7 PMC10838242

[45] J. Brejchova , K. Brejchova , and O. Kuda , “Metabolic Pathways of Acylcarnitine Synthesis,” Physiological Research 73, no. S1 (2024): S153–S163. 38752770 10.33549/physiolres.935261 PMC11412349

[46] M. Dambrova , M. Makrecka‐Kuka , J. Kuka , et al., “Acylcarnitines: Nomenclature, Biomarkers, Therapeutic Potential, Drug Targets, and Clinical Trials,” Pharmacological Reviews 74, no. 3 (2022): 506–551. 35710135 10.1124/pharmrev.121.000408

[47] S. T. Tan , T. Ramesh , X. R. Toh , and L. N. Nguyen , “Emerging Roles of Lysophospholipids in Health and Disease,” Progress in Lipid Research 80 (2020): 101068. 33068601 10.1016/j.plipres.2020.101068

[48] F. Errico , L. Gilio , A. Mancini , et al., “Cerebrospinal Fluid, Brain, and Spinal Cord Levels of L‐Aspartate Signal Excitatory Neurotransmission Abnormalities in Multiple Sclerosis Patients and Experimental Autoimmune Encephalomyelitis Mouse Model,” Journal of Neurochemistry 166, no. 3 (2023): 534–546. 37332201 10.1111/jnc.15884

