# Supplementary material for: Metabolic Signatures and Diagnostic Strategies for Different Types of Encephalitis
Source: CNS Neurosci Ther. 2026 Jul 11;32(7):e71014. doi: 10.1002/cns.71014 (PMC13354942; doi:10.1002/cns.71014)
Supplement: Supplementary file 1 — Figure S1: Permutation test results (200 permutations) for the orthogonal partial least squares‐discriminant analysis (OPLS‐DA) models. [file CNS-32-e71014-s001.docx]

**Supplementary Information**

**Metabolic Dysregulation Reveals Pathological Mechanisms and Diagnostic Strategies for Different Types of Encephalitis
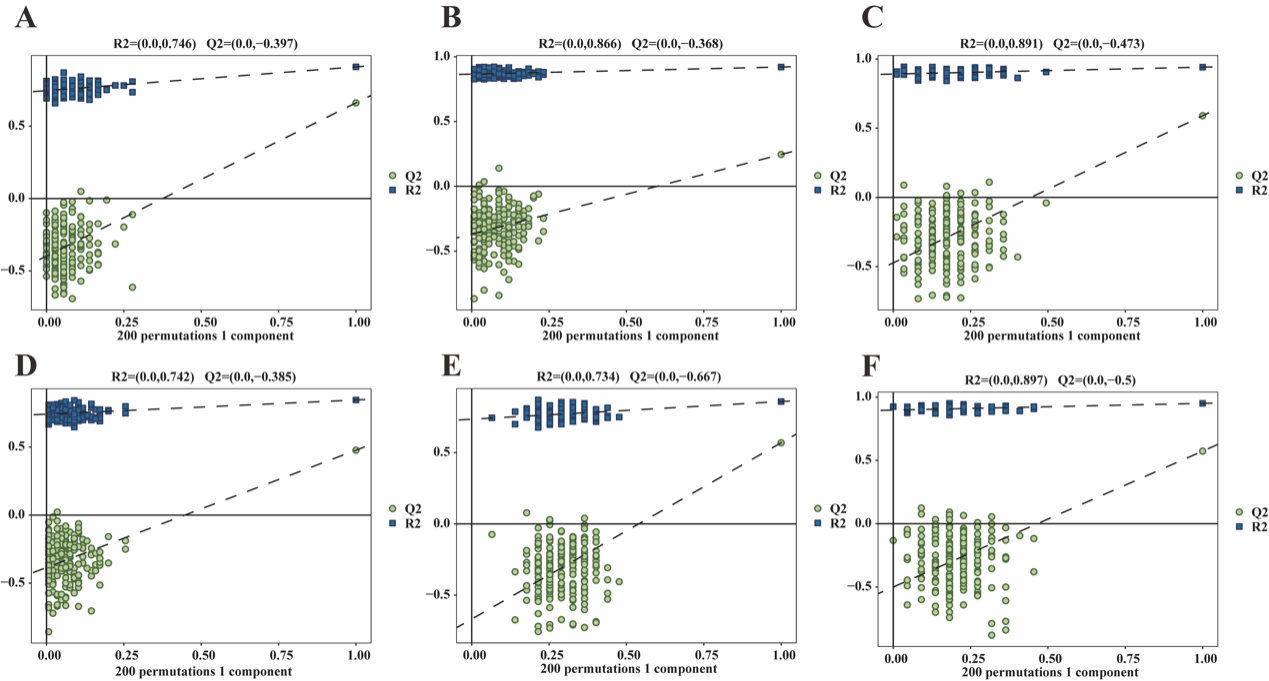
**

**Supplementary A Figure 1.** Permutation test results (200 permutations) for the orthogonal partial least squares-discriminant analysis (OPLS-DA) models. Panels (A) to (F) display the test outcomes for the following comparisons: (A) VE vs. NE, (B) AE vs. NE, (C) OIE vs. NE, (D) VE vs. AE, (E) VE vs. OIE, and (F) AE vs. OIE. AE, autoimmune encephalitis; NE, non-encephalitis disease control; OIE, other infectious encephalitis; VE, viral encephalitis.

**Supporting information for method section**

**Inclusion Criteria**

Patients were enrolled in the study if they met all of the following criteria: (1) Admitted with a suspected diagnosis of encephalitis, presenting with acute-phase clinical manifestations; (2) completion of a comprehensive diagnostic workup, including all relevant laboratory tests and ancillary investigations, as their clinical condition permitted; and (3) a final clinical diagnosis confirmed by consensus between two independent neurologists at discharge.

**Exclusion Criteria**

Participants were excluded from the study based on any of the following: (1) incomplete collection of essential demographic or clinical data; (2) failure to undergo lumbar puncture for cerebrospinal fluid (CSF) analysis due to critical illness or other reasons; (3) concurrent receipt of immunomodulatory therapy at the time of sample collection; or (4) potential co-existing central nervous system diseases that could severely confound the CSF metabolomic profile at the time of sample collection.

**Group Classification**

Prior to data extraction, we excluded participants with any of the following: an uncertain final diagnosis, multiple concurrent encephalitis, or meningitis without parenchymal involvement. The remaining participants were categorized into the following groups based on their final diagnoses:

(1) Viral Encephalitis (VE) Group

Diagnosis was established using the criteria for possible infectious or autoimmune encephalitis proposed by Venkatesan et al.[1], which requires fulfillment of the major criterion plus at least two minor criteria. The diagnosis of VE was confirmed only after systematically excluding alternative diagnoses, including:

- Non-viral infections (via CSF cultures or polymerase chain reaction)
- Immune-associated encephalitis (via comprehensive antibody testing in serum and CSF)
- Structural brain lesions (via expert-reviewed neuroimaging)
- Metabolic or toxic encephalopathies (via standardized laboratory screening)

In addition, unless the pathogen is detected in the CSF, all patients are required to show significant neurological improvement after standard antiviral treatment. Metagenomic next-generation sequencing was performed on all CSF samples to further characterize the cohort.

(2) Autoimmune Encephalitis (AE) Group

Patients were included if they met the diagnostic criteria for any AE subtype, primarily based on the 2016 criteria for definite or possible AE by Graus et al.[2] and the 2021 best practice recommendations from the Autoimmune Encephalitis Alliance Clinicians Network[3], while systematically ruling out other potential diseases. Specific criteria for subtypes were applied as follows:

- Anti-N-MethylD-Aspartate Receptor encephalitis, autoimmune limbic encephalitis, antibody-negative AE, acute disseminated encephalomyelitis, and Bickerstaff brainstem encephalitis were diagnosed according to Graus et al.[2]
- Paraneoplastic encephalitis was diagnosed using the criteria from Graus et al.[4]
- Glial fibrillary acidic protein (GFAP) antibody-associated encephalitis was diagnosed based on a combination of features, and GFAP antibodies is positive[5].
- Anti-myelin oligodendrocyte glycoprotein antibody-associated encephalitis was diagnosed according to the criteria established by Banwell et al.[6]

(3) Non-Encephalitis disease control (NE) Group

This control group comprised patients who received a definitive diagnosis for a condition other than encephalitis from a relevant specialist. These conditions included, but were not limited to: acute cerebral infarction, brain tumors, primary mental disorders and other systemic infections.

(4) Other Infectious Encephalitis (OIE) Group

This category included encephalitis types other than VE or AE, such as those caused by bacterial, fungal, tuberculous, brucella, or syphilitic infections. Diagnosis required that patients present with a clinical syndrome characteristic of the specific encephalitis, supported by either a relevant past medical history or definitive laboratory confirmation (pathogen or specific antibody detection) in blood or CSF.

Additionally, 10 patients underwent follow-up, and their second CSF samples were categorized into a separate follow-up group for data extraction.

**Ultra-Performance Liquid Chromatography-Tandem Mass Spectrometry Parameters**

Chromatographic separation was performed on an ACQUITY UPLC HSS T3 column (100 × 2.1 mm, 1.8 μm) maintained at 45°C. A binary gradient of solvent A (water containing 0.1% formic acid) and solvent B (acetonitrile) was applied at a flow rate of 0.35 mL/min over 16 min under the following profile: 0–2 min, 5% B; 2–14 min, 5% to 100% B; 14–15 min, 100% B; followed by a 1‑min re‑equilibration at 5% B.

For mass spectrometry analysis, the following parameters were applied in both positive and negative ionization modes, with the exception of spray voltage. Source conditions were as follows: sheath gas flow rate, 35 Arb; auxiliary gas flow rate, 8 Arb; auxiliary gas heater temperature, 350 °C; capillary temperature, 320 °C. Full-scan MS data were collected at a resolution of 70,000 across a mass range of m/z 70–1050. For MS/MS acquisition, a resolution of 17,500 was used with stepped normalized collision energies of 10, 20, and 40. The spray voltage was set to +3.8 kV in positive ion mode and –3.0 kV in negative ion mode.

**Statistical analysis**

**Metabolite Network Analysis**

For network analysis, group-specific weighted metabolite correlation networks were built from the union of important differential metabolites. Edges were retained only if Spearman |r| > 0.6 and Benjamini–Hochberg FDR < 0.05, yielding sparse adjacency matrices. Topological features and Walktrap-derived functional modules were computed with the NetCoMi R package. Nodes concurrently ranked among the top five for ≥3 of the four centrality indices (degree, betweenness, closeness, eigenvector) were designated as key nodes.

**Reference**

1. Venkatesan A, Tunkel AR, Bloch KC, Lauring AS, Sejvar J, Bitnun A, et al. Case definitions, diagnostic algorithms, and priorities in encephalitis: consensus statement of the international encephalitis consortium. Clin Infect Dis. 2013;57:1114–28. https://doi.org/10.1093/cid/cit458

2. Graus F, Titulaer MJ, Balu R, Benseler S, Bien CG, Cellucci T, et al. A clinical approach to diagnosis of autoimmune encephalitis. Lancet Neurol. 2016;15:391–404. https://doi.org/10.1016/S1474-4422(15)00401-9

3. Abboud H, Probasco JC, Irani S, Ances B, Benavides DR, Bradshaw M, et al. Autoimmune encephalitis: proposed best practice recommendations for diagnosis and acute management. J Neurol Neurosurg Psychiatry. 2021;92:757–68. https://doi.org/10.1136/jnnp-2020-325300

4. Graus F, Delattre JY, Antoine JC, Dalmau J, Giometto B, Grisold W, et al. Recommended diagnostic criteria for paraneoplastic neurological syndromes. J Neurol Neurosurg Psychiatry. 2004;75:1135–40. https://doi.org/10.1136/jnnp.2003.034447

5. Hagbohm C, Ouellette R, Flanagan EP, Jonsson DI, Piehl F, Banwell B, et al. Clinical and neuroimaging phenotypes of autoimmune glial fibrillary acidic protein astrocytopathy: A systematic review and meta-analysis. Eur J Neurol. 2024;31:e16284. https://doi.org/10.1111/ene.16284

6. Banwell B, Bennett JL, Marignier R, Kim HJ, Brilot F, Flanagan EP, et al. Diagnosis of myelin oligodendrocyte glycoprotein antibody-associated disease: International MOGAD Panel proposed criteria. Lancet Neurol. 2023;22:268–82. https://doi.org/10.1016/S1474-4422(22)00431-8
